# An examination of the barriers to and benefits from collaborative couple contraceptive use in Rwanda

**DOI:** 10.1186/s12978-021-01135-6

**Published:** 2021-04-19

**Authors:** Hilary Schwandt, Angel Boulware, Julia Corey, Ana Herrera, Ethan Hudler, Claudette Imbabazi, Ilia King, Jessica Linus, Innocent Manzi, Madelyn Merritt, Lyn Mezier, Abigail Miller, Haley Morris, Dieudonne Musemakweli, Uwase Musekura, Divine Mutuyimana, Chimene Ntakarutimana, Nirali Patel, Adriana Scanteianu, Biganette-Evidente Shemeza, Madi Stapleton, Gi’anna Sterling-Donaldson, Chantal Umutoni, Lyse Uwera, Madeleine Zeiler, Seth Feinberg

**Affiliations:** 1 Western Washington University, 516 High Street, MS9118, Bellingham, WA 98225 USA; 2 Spelman College, 350 Spelman Ln SW, Atlanta, GA 30314 USA; 3 Wheaton College, 26 E Main St, Norton, MA 02766 USA; 4 Northwest Vista Community College, 3535 N Ellison Dr., San Antonio, TX 78251 USA; 5 Whatcom Community College, 237 W Kellogg Rd, Bellingham, WA 98226 USA; 6 INES, Ruhengeri, Musanze, Rwanda; 7 Xavier University, 1 Drexel Dr., New Orleans, LA 70125 USA; 8 UMBC, 1000 Hilltop Cir, Baltimore, MD 21250 USA; 9 SUNY Oswego, 7060 NY-104, Oswego, NY 13126 USA; 10 Western Oregon University, 345 Monmouth Ave N, Monmouth, OR 97361 USA; 11 Eastern Oregon University, One University Blvd, La Grande, OR 97850 USA; 12 University of Kentucky, Lexington, KY 40506 USA; 13 Arcadia University, 450 S Easton Rd, Glenside, PA 19038 USA; 14 Rutgers, New Brunswick, NJ USA; 15 Drexel University, 3141 Chestnut St, Philadelphia, PA 19104 USA

**Keywords:** Rwanda, Contraception, Family planning, Male involvement, Spousal communication

## Abstract

**Background:**

Supportive male involvement is strongly correlated with contraceptive use. In Rwanda, where the contraceptive prevalence rate among married women increased from 17 to 52% from 2005 to 2010, and stagnated at 53% in 2015, understanding the role of male partners in collaborative couple contraceptive use can help inform programs designed to further increase the use of contraception in Rwanda.

**Methods:**

This study utilized qualitative methods in 2018, specifically 32 in-depth interviewers with mostly current users of modern contraceptive methods and eight focus group discussions with family planning providers—both family planning nurses and community health workers (CHWs). Respondents were from Musanze and Nyamasheke Districts, the districts with the highest and lowest modern contraceptive use, respectively, to explore the role of couple collaboration in family planning use in Rwanda. Data were analyzed using the thematic content approach in Atlas.ti (8).

**Results:**

Findings demonstrate that some men are opposed to use of male methods of contraception, and some are opposed to any contraceptive use, which can lead to covert use. Women and providers prefer collaborative couple contraceptive use—as a result, providers advocate for and encourage male partner participation in contraceptive use. Women are most often burdened with seeking out information, initiating discussions, and sharing information discovered about contraceptive use with partners. Decision-making about contraceptive use, once discussed, can be collaborative and motivated by financial considerations. When couple contraceptive use is collaborative, benefits range from marital harmony to husband’s support of sustained use through reminders about appointments, joint counseling, and support in managing side effects.

**Conclusion:**

Family planning providers at the community and clinic levels encourage collaborative contraceptive use among couples and some Rwandan couples communicate well about family planning use. Despite the positives, women are expected to source family planning information, share that information with their male partners, seek out family planning services, and use family planning. If more Rwandan male partners accepted use, used male methods of contraception, and participated even more in the work it takes to use family planning, the potential for sustained, and even enhanced, contraceptive use in Rwanda could be realized.

## Plain English summary

When male partners are involved, couples are more likely to use family planning. This study aims to understand the role of male partners in couple’s family planning use in Rwanda.

This study included 32 interviews with women who use family planning and eight group discussions with family planning providers.

The findings show that some men are opposed to use of male methods of contraception, and some are opposed to any contraceptive use. Women and family planning providers like it best when couples use family planning services and methods as a team. Women are often responsible for finding information, starting conversations, and sharing information about family planning with their male partners. Often men and women in relationships discuss and decide to use family planning together due to concerns about having enough money to care for all of their children. When couples decide to use family planning together there are many benefits. The benefits can range from support through reminders about appointments, attending services as a team, and support in managing any issues that may come up, as well as happiness in their marriage.

The current amount of male involvement in family planning in Rwanda is high for some couples, however, women still do most of the work to learn about, start, and continue family planning use. If more male partners in Rwanda became involved in family planning with their partners, and did more of the work it takes to use family planning—even more couples would likely use family planning in Rwanda.

## Background

The Programme of Action produced at the 1994 International Conference on Population and Development in Cairo, Egypt, included a focus on gender equality. This focus stated that in order to achieve more equitable reproductive health outcomes men will have to be engaged deeply as they are the ones that yield power in most spheres of life, from policy making in the government to intimate fertility decisions at the family level. The gender equality section highlighted that communication between men and women will be necessary to achieve reproductive health (1). Despite a history of awareness about the powerful role men play in family decisions [1–3], family planning program efforts have historically focused on women as the primary audience for messaging, services, and outreach [4, 5]. This focus on women persists for most contemporary family planning programs around the world [6]. In addition, most of the workers who provide family planning services are female—further contributing to the view that family planning is in the woman’s domain only [7, 8].

In patriarchal nations in sub-Saharan Africa (SSA), men make all major family decisions; however, family planning has been labeled as within the woman’s realm. In fact, in many nations in SSA men explain their lack of involvement in family planning due to the fact that it is a woman’s issue [6, 8]. Given that males make decisions about family matters yet see family planning as a woman’s purview, couples may face a difficult situation in that family planning may be needed to meet the family needs but communication between two parties about the overlapping needs might be limited or non-existent. Decisions about family size and family planning are made by two different people but ideally those decisions are synchronized. In order to bridge this gap, communication between spouses is necessary. This is likely why spousal communication about family planning is often a strong predictor of current family planning use [1, 9–12].

The availability of contraceptive methods is another area of female focus in family planning programs. The majority of family planning methods have been designed for use by the female partner, and the majority of use globally is by the female partner as the share of contraception globally attributed to male methods is around a quarter of all use [13–15]. Today there is no hormonal male method available, hence, male uptake of contraceptive use is constrained by the limited options available—vasectomy, condom, standard days method, and withdrawal [6, 16]. Vasectomy remains unpopular in SSA primarily due to misconceptions, provider bias, accessibility, and its permanence [16, 17]; however, another issue with vasectomy is that awareness about it is low, often lower than awareness of female sterilization [7].

Another male-controlled method, the male condom, is often seen as used for sexual encounters outside marriage and as such not appropriate for use within marriage [8, 18]. Along these lines, research has shown that some men believe that any woman using contraception is promiscuous [8, 17, 19]. As a result of the limited male methods available as well as the views about those methods, a potential route for male involvement in couple contraceptive use is to be a supportive partner of the female partner’s initiation and use of contraception.

Lack of spousal communication about family planning, often due to perceived spousal disapproval, has been noted by women as a major obstacle to using contraceptives [18]. A particularly strong indicator of family planning use, in low use settings, is the woman’s perception of her husband’s approval of family planning [11] as well as spousal communication about family planning use [9, 20, 21]. Research shows that when male partners are involved in family planning, women are much more likely to initiate and sustain family planning use [22, 23].

In Rwanda, the contraceptive prevalence rate among currently married women increased from 17 to 52% in between 2005 and 2010, and then nearly stalled to 53% in 2015. The total fertility rate correspondingly decreased from 6.1 to 4.6 from 2005 to 2010, and then further declined to 4.2 in 2015 [24]. The increase in contraceptive use to over half of women of reproductive age, particularly in such a short time period and in this region of the world, is unprecedented. Rwanda’s family planning program ranks very highly when compared to other family planning programs around the world [25]. In this setting of substantial gains in contraceptive uptake, it is important to study the role of male involvement in family planning to better understand the contraceptive context within couples in Rwanda. This study aims to understand the role that male partners play in collaborative couple contraceptive use in Rwanda.

## Methods

This study was conducted in Rwanda using qualitative methods. Eight focus group discussions were conducted in February of 2018 with family planning providers. Then in July of 2018, 32 in-depth interviews with mostly current contraceptive users were conducted.

The focus group discussions and in-depth interviews were evenly split between Musanze and Nyamasheke districts. These two districts in Rwanda were selected as they had the highest and lowest modern contraceptive prevalence rates among married women, respectively, as reported in the latest Demographic and Health Survey, to explore whether experiences with spousal communication about family planning use differed in the two districts [24].

The eight Focus Group Discussions (FGD) with family planning providers took place with an equal number of provider types—four with family planning nurses and four with Community Health Workers (CHW). Each village in Rwanda has three community elected CHWs, two females and one male, tasked with providing health services within their communities—including family planning services, such as: counseling, referrals to health facilities, and resupply of select contraceptive methods (condoms, pills, and injectables) [26, 27]. Each FGD had between eight and 12 participants. There were 88 family planning providers in total who participated in the study. Each FGD lasted around two hours. Family planning providers were recruited via government hospital and NGO staff who had regular contact with all family planning providers in the district.

A total of 32 In-Depth Interviews (IDI) were conducted in July of 2018 with female contraceptive users who were at least 18 years of age. Contraceptive users were recruited via family planning providers, family planning nurses at public health facilities and CHWs, as 91% of contraceptive users in Rwanda source their contraceptives from government sources [24]. The average interview duration was 43 min.

The sample size was determined prior to data collection based upon the desire to elicit information from at least two focus groups in each locale with each provider type. Originally, the study was designed to conduct sixteen interviews in each district—eight with women who were currently using modern contraceptive methods for at least 6 months and eight with women who had discontinued modern contraceptive use in the past 6 months—for a total of 32 IDIs across the two districts. It was not until data collection had already begun that the study team learned that there was miscommunication about what recent modern contraceptive discontinuers meant and that finding women who had discontinued modern contraceptive methods in the past 6 months proved to be a challenge for the family planning providers. It became clear later that recruitment of recent discontinuers was not happening as designed, and that women who were using condoms were considered discontinuers of modern methods. As a result, the data analysis and results are not disaggregated by currently using and recent discontinuers.

Male and female undergraduate educated native Kinyarwanda speakers were recruited and trained to conduct the FGDs and IDIs using topic guides. The training included an introduction to the study, review of the topic guides, best practices in qualitative data collection, role of the data collectors, data analysis in the field, and research ethics. The data collectors spent ample time with the topic guides—reading, explaining, discussing, and role-playing in preparation for the data collection.

The focus group discussions and in-depth interviews were all conducted in Kinyarwanda with only the study participant(s) and interviewer/moderator/note-taker(s) present. Every data collection activity was audio recorded with study participant permissions. Audio recordings were then translated into English and transcribed. Analysis began with every author coding the same transcript, then sharing their codes and coding decisions with the rest of the team. Then the team collated a master code list, with definitions, to guide the remaining coding work. As each team member worked through coding all remaining transcripts, the team met on a regular basis to discuss and edit the code list, if necessary, during the coding process. Coding was guided by thematic content analysis [28] utilizing Atlas.ti version 8 [29] until inductive thematic saturation across participants was reached [30]. Codes were then transferred into and analyzed in Microsoft Excel. Each team member was assigned a thematically similar group of codes to further analyze at this stage. The data were inserted into tables where the study participants made up the rows, the subthemes were the columns, and the cells were the direct quotes. At this stage subthemes were further read, and re-read, and analyzed for additional subthemes, or combined if complementary—and then drafted into narrative sections with example quotes. The lead author oversaw and reviewed all data analysis work and output.

Ethical approval to conduct this study was obtained from institutional review boards at Western Washington University and at the Rwandan Ministry of Education. Every study participant read and signed a written consent form prior to participation. Study participants were compensated 10,000 Rwandan francs for their transportation costs and time (~ $10 USD).

This research was supported by a National Science Foundation grant under the funding stream of Research Experience for Undergraduates. This paper includes findings from two different sets of undergraduate students from the USA and Rwanda who conducted the research under the tutelage of two professors.

## Results

### Demographics of the sample

There were 88 family planning providers participating in the FGDs. Just over half were CHWs at 54% of the sample. Most family planning nurses were female, 95%, while nearly half of the CHWs were male, 48%. The age of providers ranged from 28 to 61, with an average of 44 years of age. Among the 32 female contraceptive users, the average age was 38 years, ranging from 26 to 50. Over 90% of the sample was married. On average, the contraceptive users had four children, ranging between one and nine children. Contraceptive methods used included: condoms, pills, injectable, implants, and sterilization. Injectable was the most common method used. Average use duration of the current method was 5 years.

### Organization of themes

The findings fell into four main overarching themes: (1) Barriers to Collaborative Couple Contraceptive Use, (2) Addressing Barriers to Collaborative Couple Contraceptive Use, (3) Progression of Couple Communication and Decision-Making about Contraceptive Use, and (4) Benefits Derived from Collaborative Couple Contraceptive Use.

## Barriers to collaborative couple contraceptive use

Within the theme of Barriers to Collaborative Couple Contraceptive Use, two subthemes emerge: male method use and covert use*.*

### Male method use

Contraceptive users noted a desire to share the side effect burden with their male partners; however, the only modern male method that was acceptable to most of the male partners was the condom.

My husband doesn’t want to use family planning, he says, “No no, I can’t go to use family planning, it’s impossible for me.”

Female, 42, injectable user, 3 children, Musanze, IDI

In the village, women are the ones who understand more about using contraceptives. Men do not want to hear about or to use contraceptives, because the men don’t accept the methods that are available to them.

Female, 41, injectable user, 5 children, Musanze, IDI

I tried to discuss with my husband about him using male family planning methods so I could use it for 3 months, then he could use his for 3 months and we rotate like that. But he said no, and he refused to go to the hospital to learn about possible methods for himself.

Female, 38, injectable user, 5 children, Musanze, IDI

Some discussions with the family planning users surrounded method specific choices—in particular, how couples negotiated method choice when male methods are an option.

My husband doesn’t want to get sterilized, but he agreed with me that I should get sterilized.

Female, 40, sterilized, 9 children, Nyamasheke, IDI

Raised also only by CHWs in Musanze was a plea for more male contraceptive methods.

We wish that men could also have their own method. Even if it is for a year it is ok because then the women will be able to take a break…

CHW, male, 48, Musanze, FGD

### Covert use

The topic of covert use arose in almost all focus group discussions with providers. The main message conveyed was that covert use does exist when necessary. Nurses noted this theme more frequently than CHWs.

It is a possibility that her husband may not agree on the use of contraceptives. However, that does not mean that she is not allowed to utilize family planning services. She may ultimately decide to use contraceptive methods without her husband’s support.

Nurse, male, 40, Musanze, FGD

Furthermore, when women resort to covert use, providers’ words demonstrated how they would work with women to use contraceptives in a manner that aligned with their needs and did not expose their use.

The CHW will look for her partner and teach him and he won’t show (tell) the husband that his wife has started using family planning.

CHW, male, 42, Musanze, FGD

Women noted that the challenges with covert use involved husbands discovering and stopping her use of family planning as well as women receiving subpar services due to their fear of being discovered. Additionally, some women noted how contraceptive use could lead to problems in the marital relationship if not done as a team.

…I used to talk to my husband about it and he did not understand it well. And I thought that if I used family planning, it might lead to divorce and break the family.

Female, 43, injectable user, 4 children, Nyamasheke, IDI

Although rare, some women interviewed chose to not initially disclose their decision to use family planning methods with their husbands because the husbands were not agreeable about the use of family planning but were planning to disclose to their husbands over time. Some husbands who were initially disapproving of family planning changed their minds once they saw the advantages, and through frequent dialogue with their wives, they became supportive. All of the respondents who noted initially opposed partners reported that their spouses eventually supported and accepted their decision to use contraception—but this was among this sample, respondents unsuccessful in their efforts to do the same were likely not included in the sample. This theme arose more in Nyamasheke than in Musanze.

The first time I told my husband I wanted to use injectables he didn’t understand it but after a while he started understanding how important it is and we don’t have any problems about him.

Female, 40, sterilized, 9 children, Nyamasheke, IDI

## Addressing barriers to collaborative couple contraceptive use

Two subthemes arose in the Addressing Barriers to Collaborative Couple Contraceptive Use theme, they are: encouraging male involvement in family planning and encouraging spousal communication about family planning.

### Encouraging male involvement in family planning

A few participants noted that community meetings, such as: Umaganda and Akagoroba K`Ababyeyi, were an opportune time to encourage men to support their wives in family planning use.

…they have to encourage the men to learn about contraceptives, because there are women who want to use contraceptives, but their husbands won’t allow them to go and seek contraceptive methods. To me, I think it would be good if they gave advice in Umaganda and in Akagoroba K`Ababyeyi, because the men also come to the Akagoroba K`Ababyeyi meeting.

Female, 45, pill user, 2 children, Nyamasheke, IDI

Others noted how home visits could help men change their initially oppositional views.

If a husband doesn’t want his woman to go and use contraceptives, the community health workers go to the family and try to teach the husband and explain to him why contraceptives are important.

Female, 38, pill user, 3 children, Musanze, IDI

CHWs in Musanze and contraceptive users also brought up the role that CHWs can play in encouraging male involvement in family planning.

There are some women who are interested in using family planning but their partner or husband is not interested or aware of it so the advice I can give them (CHWs) is that they have to visit the houses who are in that situation so that they can convince both sides.

Female, 32, injectable user, 2 children, Nyamasheke, IDI

…there are families that feel like using family planning is for women only. It’s my understanding that as CHW it is my job to sensitize people. In our way of advising, we also have to work with courage so that we can sensitize people who have not understood yet. The CHW has to sensitize the family themselves by telling them that deciding to use family planning is the job of the both the husband and wife.

CHW, female, 37, Musanze, FGD

### Encouraging spousal communication about family planning use

Despite supporting covert use when necessary, family planning providers were concerned about how the use of family planning, without consent of the husband, would result in challenges for the household. Family planning providers shared how women must discuss the topic of family planning with their spouse prior to initiating contraceptive use. They presented the onus of spousal communication as falling upon the woman’s shoulders.

I would discuss and ask if she has already talked about it with her husband so that she will not have any problems with him.

CHW, male, 61, Musanze, FGD

Family planning providers and contraceptive users both noted how family planning use is most successful when husbands and wives communicate about use prior to initiating use.

I would advise her not to use contraceptive methods if she has not talked about it with her husband first…

CHW, female, 61, Musanze, FGD

## Progression of couple communication and decision-making about contraceptive use

In the Progression of Couple Communication and Decision-Making about Contraceptive Use theme, there are four subthemes: initiation of spousal discussion, coming together for counseling is best, decision-making about contraceptive use, and financial considerations.

### Initiation of spousal discussion

Most often, the sequence narrated by family planning providers was that the woman would be exposed to family planning messages—at the health center, by a CHW, or through another source. Once she was informed about family planning, and interested in pursuing the use of a method, the next step for her would be to go back home to discuss the decision with her husband.

After she gets information about family planning, she discusses it with her husband because she can’t make the decision alone.

Nurse, female, 55, Nyamasheke, FGD

Less often the sequence noted by providers was that the woman discusses the topic of initiating family planning use with her husband first, and then seeks out more information from a provider. Family planning providers in Musanze more often noted this than providers in Nyamasheke.

…she must first come to a mutual agreement with her husband. Afterwards, she can go to family planning provider at a health center or to a nurse.

Nurse, female, Musanze, FGD

Women reported that when the discussion about family planning was brought up by one partner, it was more likely to be initiated by the female partner; however, some participants noted that their male partner was the one to initiate the discussion about family planning in the household.

I: When you first discussed that idea of family planning to your husband, how did he feel?

R: I was so surprised because it was he who first told me about family planning…

Female, 38, condom user, 2 children, Nyamasheke, IDI

### Coming together for counseling is best

Providers consistently noted how couple counseling is the best, not only for them, but for the couples as well. This theme arose more often among nurses and providers in Musanze than among CHWs and Nyamasheke providers.

It’s better if she comes together with her husband and you tell them the good of using family planning and the bad of kids close in age.

CHW, female, 44, Musanze, FGD

If she comes with her husband, it would be easier. If you explain to her and she doesn’t understand or she might say she has to go back home to discuss with her husband. If they come together, it will be easier because they make their decision at that time in that place.

Nurse, female, 40, Musanze, FGD

In situations where the men were not present, the onus was placed upon the woman to ensure her male partner participates in the process in the future. Furthermore, if they do not come as a couple, the woman must come for the information, return home to discuss with her husband, and then sometimes go through that process again when deciding upon a method.

When it comes to the decision of family planning there are two people (i.e. husband and wife) who need to be informed. Ultimately it is the women’s choice; however, if she doesn’t possess the confidence or put in the effort, her husband may not be convinced.

Nurse, male, 40, Musanze, FGD

### Decision-making about contraceptive use

Around three-quarters of the women described discussions and decisions about family planning with their husbands as collective, meaning that the decision was not just made by the husband or wife but, instead, as a union based upon prior communication. Collective decision-making was slightly more often noted in Nyamasheke than in Musanze.

I: Can you tell me how your husband felt about the decision to use family planning?

R: My husband received this decision well because we took much time to discuss it. And if you discuss with your husband about it, and you both agree, there is no problem.

Female, 38, implant user, 5 children, Nyamasheke, IDI

I: Can you tell me how your partner felt about this decision (to start using family planning)?

R: There was no problem, because we had to discuss it before I went to use family planning. He supports me because he doesn’t want me to have more kids than we can take care of.

Female, 41, injectable user, 5 children, Musanze, IDI

### Financial considerations

Over two-thirds of the women reported their discussions about family planning were related to having the financial means to properly manage family needs. Incorporating topics about family management and being able to support your family was a reoccurring theme in many of the spousal conversations about family planning. Inclusion of family management in spousal discussions of family planning use was more common in Nyamasheke than in Musanze.

I: You told me that you recently used family planning; can you tell me how your husband felt about this decision?

R: My husband received this decision well because he saw how life was hard and we discussed it together saying let’s use family planning so that our children can grow up well. We said we’d wait to have another child when our life got better.

Female, 38, condom user, 4 children, Nyamasheke, IDI

## Benefits derived from collaborative couple contraceptive use

There are two subthemes, marital stability from contraceptive use and male support can extend beyond communication, in the Benefits Derived from Collaborative Couple Contraceptive Use theme.

### Marital stability from contraceptive use

While some study participants shared how divorce can occur from covert use, even more noted how the lack of contraceptive use can also lead to problems, such as divorce, for married persons.

I use family planning because I don’t want fights in my family.

Female, 38, pill user, 3 children, Musanze, IDI

…it helped my family because if you have more kids it can also create a dispute with you and your husband because you see that there is a number you can handle together. There are ones who have this problem and it causes them to get divorced. All these things won’t happen because we use contraceptives.

Female, 45, pill user, 2 children, Nyamasheke, IDI

Years ago, men in this village didn’t have any knowledge of contraceptives, and if they heard that if a woman goes to use contraceptives it would cause a dispute in the family because the husband didn’t want it. But now there is not a man with that kind of belief. Now the men mostly go with the women when they want to get contraceptives.

Female, 38, pill user, 3 children, Musanze, IDI

### Male support can extend beyond communication

Women noted how spousal support often extends beyond verbal agreements. Many husbands played influential roles in supporting their wives on their journey with contraceptive method use in terms of motivating and supporting use—especially through experience of side effects. These themes arose more often in Nyamasheke than in Musanze.

One thing that motivates me is that my husband continues to encourage me to use family planning.

Female, 29, implant user, 2 children, Nyamasheke, IDI

…my neighbor was also asking how we were able to space our kids, I used to tell them that we planned together and my husband helped me to use family planning so we can raise our kids that are not close in age.

Female, 38, condom user, 2 children, Nyamasheke, IDI

One of the ways husbands supported their wife’s use of family planning was through reminders about appointments with providers. Husbands could also support their wives by joining their wives for family planning counseling.

I: How did your husband feel about your decision to start using family planning?

R: My husband feels good and now he tries to go with me to the health center to get the methods and he doesn’t want to have many children, so he agrees with me. He doesn’t want me to stop using family planning for a long time. Now he tries to tell me to find many specialists so that they can help in order to continue using family planning.

Female, 36, implant user, 3 children, Nyamasheke, IDI

I: Can you tell me how your partner reacted to your decision to use contraceptives?

R: For me, my partner is the one who brought up the idea of using contraceptives because he understood the importance of them. We went together when I went to test for pregnancy and they taught us together how to use contraceptives and the importance of using contraceptives. After we gave birth, my partner told me that, since we already have a child, that he didn’t want kids close in age and that we should to think about using contraceptives so that we could have another kid when the first is grown. We sat together and discussed about using contraceptives, and I discussed with him and accepted using contraceptives.

Female, 45, pill user, 2 children, Nyamasheke, IDI

## Discussion

This study sought to better understand the role of male partners in collaborative couple family planning use in Rwanda from the perspective of family planning providers and experienced female modern contraceptive users—who were on average married, older (38 years), and had four children. While differences between the two districts, provider type, and between providers and contraceptive users did arise within a few subthemes, overall, there were more similarities than differences.

The family planning providers and contraceptive users described most male partners as involved in the family planning process in Rwanda via participation in conversations about initiating contraceptive use, continuing support of family planning use, and via condom use. Male partner use and support of female partner contraceptive use was seen as positive in terms of aiding couples in initiating and sustaining contraceptive use to meet familial spacing and limiting goals designed to support the livelihood of their families.

Providers and women in Rwanda agreed that family planning use is easiest when both partners are on the same page about the decision and go for counseling and services as a couple. If a couple decides to use a female-designed contraceptive method, male partner support of this use can range from initiating the discussion about family planning use to participating in decisions about when to initiate method use and what method to use. Male partners also support their female partner’s sustained use of contraceptives by providing general support when side effects do occur and engaging in discussions about switching methods when side effects are unbearable. In contrast, other research with men has shown how that some men are frustrated by contraceptive side effects due to how they impact sexual frequency, and therefore, discourage contraceptive use by their female partners [6, 17].

The burden of initiating discussions about contraceptive use, as well as use, was not equally shared by couples, however. Providers placed the responsibility on women to initiate discussions about family planning use with their partners—and to seek out information about family planning and to relay that information to their male partners. Most women reported that they were more likely to initiate the family planning discussion than men. The consequences of using family planning without consent, as well as not using family planning at all fell on the women and they were severe—there could be rifts in the marriage, and ultimately, even divorce.

Rwandan men were often compelled to participate in conversations and decisions about family planning use due to consideration about the ability of the couple unit to manage their finances to properly raise and care for their children. Other research in sub-Saharan Africa has also found that the financial association between family planning use and family health to be motivating for husbands to discuss contraceptive use [10, 19, 31–33].

In comparison to other family planning programs in other nations, male support of family planning use in Rwanda is unique. Male support of family planning in Rwanda might occur due to the political support for family planning at all levels of government [34–36]. Additionally, CHWs are important players in the family planning program in Rwanda—and a third of them are men. Electing fellow male community members into a role of educating and advocating for family planning use in the community, and at the household level, might also contribute to shifting norms to engage males in the family planning conversation [34]. Efforts to increase contraceptive use through a male peer education model significantly increased contraceptive use through increased spousal dialogue about family planning [10]. Research with men indicates that including them in the counseling in the home with CHWs may encourage their involvement throughout the process [19, 37]. In this study, CHWs were noted as persons who could sensitively talk with resistant men about the benefits of contraceptive use.

Despite the positives, men were still reportedly reluctant to seek out and use male methods, particularly vasectomy, similar to findings in other settings [6, 15]. However, some were reportedly using condoms with marital partners, as opposed to other research findings where men view condom use as only appropriate for extramarital affairs [18]. Women and providers noted a desire for more men to engage more fully in family planning, as others have noted as well [33, 38].

Some male partners in the community were still not supportive of their wives using family planning services. In these cases, providers discussed covert use—as necessary to support when male partners were barriers to desired contraceptive use. Alongside covert use, study participants recommended that further outreach via community meetings and home visits led by providers may be beneficial. Researchers have noted how efforts to target and involve males in family planning programs have been rare, to nonexistent [4, 7, 39]. As a result, researchers have advocated for more mobilization efforts at the individual, couple, and community levels to increase communication between spouses about fertility goals and family planning use [5, 20, 39]. There are risks, however, to increasing male involvement in family planning efforts that must be thoroughly considered throughout any program design and implementation. Any intervention designed to increase male involvement in family planning must be sensitive to these risks, such as reinforcing male control over female bodies and decisions, stigmatizing women who seek out family planning without their male partner, and denying services to women who seek out contraceptive services without their male partner present or without his consent—some of which arose in this study, particularly by providers. Furthermore, interventions in this arena must be gender transformative as opposed to gender blind, while also being culturally sensitive, in that they should seek to shift gender relations by challenging current gender norms that reproduce gender inequity on an individual, family, community, and structural level [39–41].

This study had a few limitations. Most importantly, male partners were not included in the sample so views about male involvement in the family planning process were only sourced from female partners and family planning providers. Mostly current contraceptive users were included—so women who have never used family planning and those who have discontinued contraceptive use while still in need were excluded from the sample. Family planning providers recruited family planning users, so the sample was likely biased towards satisfied users. In addition, analysis of the sample demographics showed that the sample was older, at 38 years, married, with an average parity of four. Translation and transcription occurred in the same step—whereas transcribing first in Kinyarwanda and then translating into English would have increased accuracy of translation. Finally, this study occurred in just two districts in Rwanda, and is qualitative, so the results are not generalizable.

The strengths of this study were the triangulation of data in that both contraceptives users and family planning providers were included in the study—and similarities as well as inconsistencies between the two samples were examined. The family planning provider sample also included both family planning nurses and community health workers—to increase the perspectives included from the providers who work with clients in the clinic and those who work more intimately in the community with their neighbors.

Future research in this area should include male partners in the study to understand couple dynamics around family planning use in Rwanda from the male perspective. Additionally, research that is inclusive of couples who have not used contraception or have used and discontinued use despite still wanting to avoid pregnancy, would help fill gaps in understanding collaborative couple family planning use in Rwanda.

## Conclusion

Couple dynamics surrounding family planning use in Rwanda appear to be very positive for some couples. Most women in this study report communicating with their male partners about the potential of family planning use in a joint manner. The support of male partners extends beyond the initial discussion and decision phase, into the experience of interacting with providers, reminders about appointments, accompaniment to appointments, as well as motivation and support to sustain use through the unpleasant experience of side effects. However, some males remain opposed to family planning or are disengaged in the process. Study participants recommended more community meeting exposure as well as home visits in these scenarios. In addition, while family planning providers encourage male involvement in family planning, they continue to expect women to gather information, initiate discussions, and share that information with their male partners. While the current level of support some husbands are providing to their female partners in Rwanda is promising, further increasing male involvement in contraceptive use via gender transformative approaches in the future would be beneficial for Rwandan family planning providers, men, and women.

## Data Availability

The transcripts analyzed for the current study are available from the corresponding author on reasonable request.

## Abbreviations

CHW: Community Health Worker
FGD: Focus Group Discussion
IDI: In-depth interview
SSA: Sub-Saharan Africa
USD: United States Dollar
